# The density of metastatic lymph node as prognostic factor in squamous cell carcinoma of the tongue and floor of the mouth

**DOI:** 10.1590/S1808-86942012000300015

**Published:** 2015-10-14

**Authors:** Ali Amar, Abrão Rapoport, Otávio Alberto Curioni, Rogério Aparecido Dedivitis, Cláudio Roberto Cernea, Lenine Garcia Brandão

**Affiliations:** a PhD in the Otorhinolaryngology and Head & Neck Surgery Graduate Program at the Federal University of São Paulo (MD, Surgeon in the Department of Otorhinolaryngology and Head & Neck Surgery at the Heliópolis Hospital HOSPHEL).; b Professor in the Department of Surgery of the Medical School at the University of São Paulo (MD, Surgeon in the Department of Otorhinolaryngology and Head & Neck Surgery at the Heliópolis Hospital HOSPHEL).; c PhD in the Pathology Graduate Program of the Medical School at the University of São Paulo (Head of the Department of Otorhinolaryngology and Head & Neck Surgery at the Heliópolis Hospital HOSPHEL).; d Professor at Fundação Lusíada UNILUS (MD).; e Associate Professor in the Department of Head & Neck Surgery of the Medical School at the University of São Paulo (Associate Professor in the Department of Head & Neck Surgery of the Medical School at the University of São Paulo).; f Professor in the Department of Head & Neck Surgery of the Medical School at the University of São Paulo (Professor in the Department of Head & Neck Surgery of the Medical School at the University of São Paulo). Departamento de Cirurgia de Cabeça e Pescoço e Otorrinolaringologia do Hospital Heliópolis HOSPHEL, São Paulo/SP; e Departamento de Cirurgia de Cabeça e Pescoço da Faculdade de Medicina da Universidade de São Paulo, São Paulo/SP.

**Keywords:** lymph nodes, lymphatic metastasis, mouth neoplasms, prognosis, tongue neoplasms

## Abstract

The presence of metastatic lymph nodes is a relevant prognostic factor in oral cancer.

**Objective:**

This paper aims to assess metastatic lymph node density (pN+) in patients with tongue and floor-of-mouth squamous cell carcinoma (SCC) and the association of this parameter with disease-free survival (DFS).

**Materials and Methods:**

A group of 182 patients seen between 1985 and 2007 was included, 169 of which were males. Five were on stage I, 35 on stage II, 56 on stage III, and 85 on stage IV. Median values were considered in lymph node density assessment, and the Kaplan-Meier curve was used to evaluate DFS; survival differences within the group were elicited through the log-rank test.

**Results:**

An average 3.2 metastatic lymph nodes were excised from the patients in the group. Density ranged from 0.009 to 0.4, with a mean value of 0.09. Five-year DFS rates were of 44% and 28% for the groups with lymph node densities below and above the median respectively (*p* = 0.006). Two-year local/regional control was achieved for 71% and 49% for the patients below and above the median density respectively (*p* = 0.01). In terms of pN staging, local/ regional control was achieved in 70% and 54% of pN1 and pN2 patients respectively, albeit without statistical significance (0.20%).

**Conclusion:**

Lymph node density may be used as a prognostic indicator for tongue and floor-of-mouth SCC.

## INTRODUCTION

Neck clearance is a standard procedure used to stage and treat regional metastases of upper respiratory and digestive tract malignant tumors. Metastatic lymph nodes are among the most relevant findings in diagnosing advanced disease (stages III and IV). The TNM staging system is the main prognostic indicator in use today, but recent studies have pointed out that lymph node density allows for better prognostic accuracy in pN+ patients^1^, ^2^.

This study looks into lymph node density in tongue and floor-of-mouth epidermoid carcinoma patients while considering local/regional control and survival.

## MATERIALS AND METHODS

This study was approved by our institution's Research Ethics Committee and granted permit 071/2000.

This study was based on reviews performed on the charts of tongue and floor-of-mouth epidermoid carcinoma patients submitted to neck clearance between January of 1985 and December of 2007. A group of 182 patients with lymph node metastases confirmed by histopathology and staged as pN1 and pN2 was selected from a larger set of 440 patients. One hundred and sixty-nine were males and 13 were females, with a mean age of 52 years (27-78). All patients were diagnosed with epidermoid carcinoma; 77 had primary tongue tumors, 98 had floor of mouth tumors, and 6 had both sites involved. Five were diagnosed as having stage I tumors, 35 had stage II disease, 56 were on stage III, 85 on stage IV, and one patient could not be re-staged. Neck clearance was performed electively in 64 cases and with therapeutic purposes in 118 patients. Unilateral neck clearance was done in 112 patients and bilateral clearance was performed in 70 cases; 181 of the 252 clearance procedures were of the radical type and 71 were selective. Lymph nodes were individualized immediately after the procedure by one of the team's surgeons and sent to the pathologist for further examination.

Lymph node density was calculated by dividing the number of metastatic tumors by the total number of removed lymph nodes regardless of the type of clearance procedure. The median value for lymph node density was used to divide patients into two groups. Local/regional control and disease-free survival were analyzed using the Kaplan-Meier estimator and the differences between both groups were assessed through the log-rank test. Differences with an alpha error under 5% were considered significant.

## RESULTS

**Figure 1:**
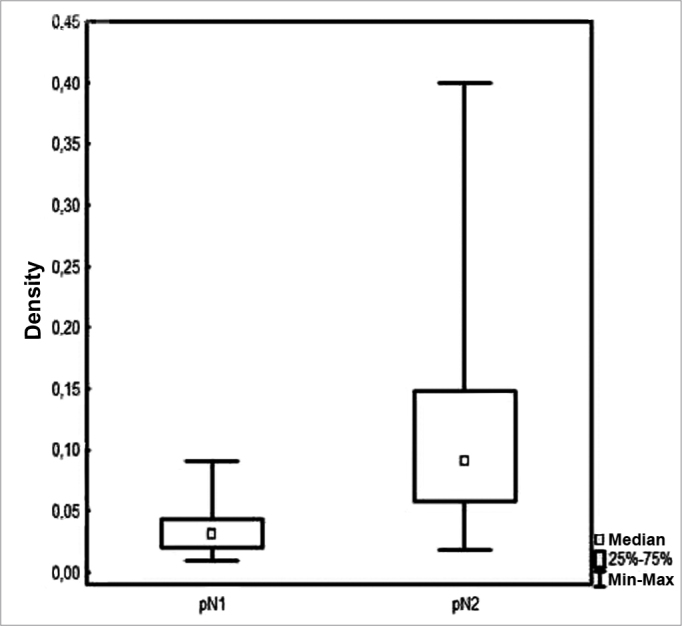
Lymph node density in accordance with pN staging.

Patients had a mean 3.2 (1-12) involved lymph nodes; 42 (7-140) lymph nodes were removed on average per patient. Density ranged between 0.009 to 0.4 with a mean value of 0.09 and a median value of 0.06 (Figure 1).

Fifty-seven patients had either local, isolated, or associated recurring tumors. Regional recurrence was seen in 32 cases; 16 were related to the primary tumor site and 10 were isolated recurring tumors. Sixteen patients were diagnosed with a second primary tumor. Distant recurring tumors were seen in 14 cases; isolated recurring tumors were seen in only 8 patients.

**Figure 2:**
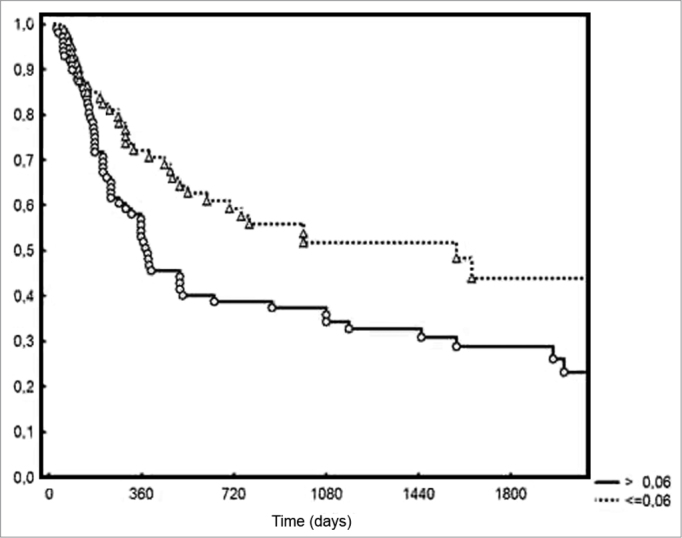
Disease-free survival for patients with lymph node density below and above the median.

**Figure 3:**
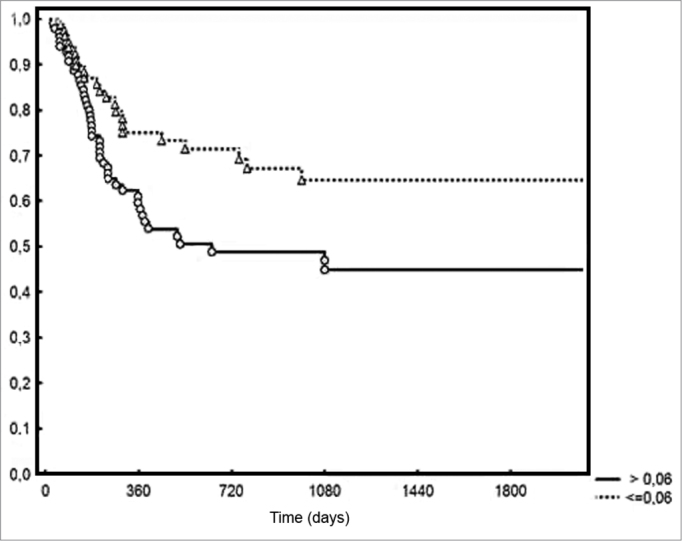
Local/regional control based on lymph node density.

**Figure 4:**
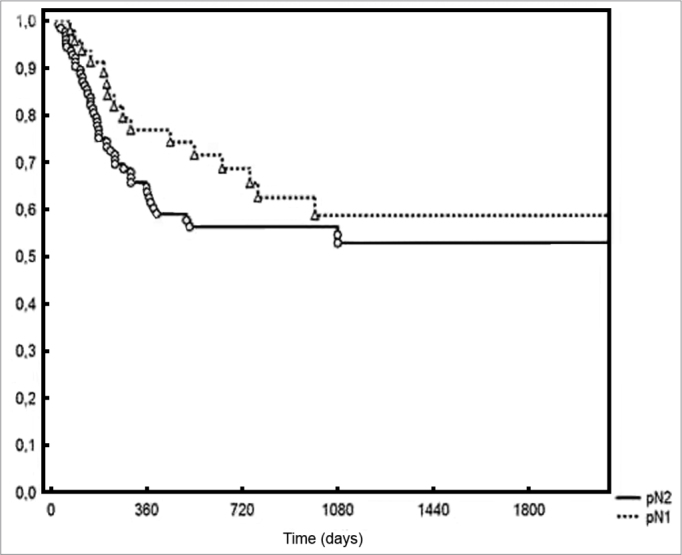
Local/regional control based on pN staging.

Five-year DFS was observed in 44% and 28% of the patients below and above the median value respectively (*p* = 0.006 - Figure 2). Two-year local/ regional control was seen in 71% and 49% of the patients below and above the median value respectively (*p* = 0.01 - Figure 3). Local/regional control was seen in 70% and 54% of pN1 and pN2 patients respectively, but failed to achieve statistical significance (*p* = 0.20 - Figure 4).

**Figure 5:**
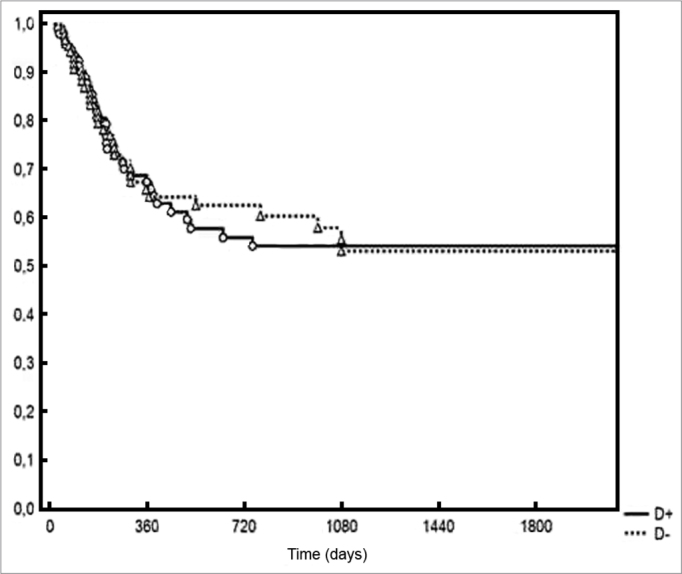
Local/regional control based on median lymph node density at the various pN stages.

Median lymph node density was 0.03 on pN1 patients and 0.09 on pN2 patients. No significant difference was observed between patients above or below the median in regards to pN staging and local/ regional control (Figure 5).

## DISCUSSION

Although neck clearance is a standard procedure, the number of retrieved lymph nodes varies dramatically in individual basis. The figures on lymph node density, number of removed lymph nodes and metastatic nodes seen in this study are consistent with other previously published series, thus indicating the similarity in the procedures carried out at various centers and in the manifestation of this disease in different populations.

The standard approach for oral epidermoid carcinoma patients is resecting the primary tumor with the proper safety margins to ensure good local control and performing neck clearance for the purposes of achieving good regional control. Radiotherapy and chemotherapy have been used in patients with later stage tumors. Adjuvant therapy may introduce toxicity and severe side effects. A significant challenge, therefore, lies in finding a reliable method to stage patients for risk of tumor relapse immediately after surgery^3^. Analysis of failure patterns in oral cancer patients reveal that approximately 33% of such cases occur due to regional metastases. One of the most significant prognostic factors in this population is the presence of neck metastasis^4^. The TNM staging system uses the number, side, and laterality of positive lymph nodes to categorize the severity of lymph node disease.

Some authors claim that the number of removed lymph nodes carries prognostic value in pN0 patients, to suggest a more accurate staging or a more responsive immune system^5^, ^6^, ^7^, ^8^, ^9^. The presence of metastatic lymph nodes is one of the most significant prognostic indicators in malignant had and neck tumors. Lymph node metastasis lead to a reduction of approximately 50% on survival, and prognosis is significantly worse when three or more metastatic lymph nodes are found^10^, ^11^.

The length of the neck clearance procedure, the surgical approach, and the level of histopathological investigation determine the degree to which lymph nodes are examined for metastasis detection and, thus, the likelihood of finding metastases in the lymph nodes at risk^6^. Thus, it is expected that these factors determine the pN lymph node stage (negative or positive).

Lymph node density (number of positive lymph nodes divided by the total number of excised lymph nodes) has recently emerged as an alternate staging system to predict survival after surgery in bladder and esophageal carcinoma patients. In such system, the ratio of positive lymph nodes in relation to the total number of excised lymph nodes was deemed better than the TNM staging system to predict survival. Just as neck clearance may lead to a better understanding of the severity of lymph node disease, lymph node density tries to compensate for this factor as it retrieves two bits of information: extent of neck involvement (number of positive lymph nodes) and extent of surgical eradication of such lymph nodes (total number of lymph nodes removed during surgery)^1^.

Lymph node density, in the established cut-off point, allowed the identification of a group of patients that encompasses most pN1 patients and a few pN2 patients in whom the disease has been controlled more effectively. Lymph node density is a quantitative variable, and does not appear to have any direct association with disease aggressiveness. However, it allows for better discrimination of pN+ patients with a more favorable prognosis who may not require adjuvant treatment. A density of 0.06 accounts for patients with at least 17 lymph nodes dissected for each metastatic lymph node, thus reflecting a more accurate staging system given the larger sample of resected lymph nodes.

It has been postulated that lymph node density may have significant prognostic value as it takes three factors into account: tumor (number of positive lymph nodes), therapy (number of lymph nodes removed in the neck clearance procedure), and staging (how radical the procedure was in relation to the surgeon and the pathologist)^1^. The detection of small metastatic deposits in lymph nodes may be enhanced with sequential cross sections, immunohistochemistry and/ or complementary molecular assays ^12^.

Survival outcomes in this sample reflect a group made up of pN+ patients only, 80% of whom with stage IV disease. The main reason for treatment failure was primary tumor recurrence. Isolated regional recurrence was rare.

Metastatic lymph nodes and lymph node density also reveal a phenotype of systemic disease and aggressive involvement in the primary tumor site. Lymph node density may be used as a prognostic indicator in tongue and floor-of-mouth carcinoma patients, as it is better than pN staging for pN1 and pN2 patients.
